# Commentary: Mapping the landscape and exploring trends in macrophage-related research within non-small cell lung cancer: a comprehensive bibliometric analysis

**DOI:** 10.3389/fimmu.2024.1461066

**Published:** 2024-10-02

**Authors:** Kun Xu, Hua Zhao, Ziqi Zhao, Xisheng Xu, Yuejun Zhou

**Affiliations:** School of Basic Medicine, Zhejiang Chinese Medical University, Hangzhou, Zhejiang, China

**Keywords:** macrophage, NSCLC, bibliometric, CiteSpace, VOSviewer, comment

## Introduction

A study titled “Mapping the landscape and exploring trends in macrophage-related research within non-small cell lung cancer: a comprehensive bibliometric analysis” was published in *Frontiers in Immunology* by Yinxue Zhou et al. (1). The authors utilized advanced bibliometric tools (CiteSpace and VOSviewer) for bibliometric and visualization analysis, which not only examined the research progress and hotspots in this research field, but also predicted future research trends. We highly support and appreciate the work of the researchers and thank them for the results they have achieved. Nevertheless, we have some concerns about their results and data.

Under the subheading of Analysis of cited reference and reference burst, we found three problems. First, the authors mentioned that “followed by the article published in Clinical Research, titled “Antibody-Fc/FcR Interaction on Macrophages as a Mechanism for Hyperprogressive Disease in Non-small Cell Lung Cancer Subsequent to PD-1/PD-L1 Blockade”. However, according to Table 4 in the original article, we found that the article titled “Antibody-Fc/FcR Interaction on Macrophages as a Mechanism for Hyperprogressive Disease in Non-small Cell Lung Cancer Subsequent to PD-1/PD-L1 Blockade” was published in Clinical Cancer Research. In short, “Clinical Research” should be modified to “Clinical Cancer Research”. Second, the authors mentioned that “These 25 papers experienced citation bursts between 2008 and 2023, with six of them continuing to show burst activity up until the end of the study period”. However, according to Figure 6 in the original article, we found that 8 of the 25 papers continued to show burst activity up until the end of the study period, namely “Siegel RL, 2017”, “Mantovani A, 2017”, “Rittmeyer A, 2017”, “Gordon SR, 2017”, “Rakaee M, 2019”, “Herbst RS, 2018”, “Gandhi L. 2018”, “Lambrechts D, 2018”. Third, we believed that the begin time and end time of the citation burst of the reference “Anderson JC, 2020” in Figure 6 of the original text are incorrect. Figure 6 showed that the begin time of the reference was 2020 and the end time was 2016. It can be seen that the begin time was later than the end time.

Finally, we reiterate our appreciation for the excellent work of Zhou et al., which not only helps researchers provide strategic insights, but also points the way for future NSCLC research. However, for the sake of the rigor and scientificity of the article, the author needs more rigorous expression.
